# A New 3-Day Standardized Eyeblink Conditioning Protocol to Assess Extinction Learning From Infancy to Adulthood

**DOI:** 10.3389/fnbeh.2020.00135

**Published:** 2020-08-14

**Authors:** Carolin Konrad, Dirk Adolph, Jane S. Herbert, Lina Neuhoff, Cornelia Mohr, Julie Jagusch-Poirier, Sabine Seehagen, Sarah Weigelt, Silvia Schneider

**Affiliations:** ^1^Faculty of Psychology, Clinical Child and Adolescent Psychology, Mental Health Research and Treatment Center, Ruhr University Bochum, Bochum, Germany; ^2^Wollongong Infant Learning Lab, School of Psychology and Early Start, University of Wollongong, Wollongong, NSW, Australia; ^3^Abteilung für Kinderschutz, Vestische Kinder- und Jugendklinik Datteln, Universität Witten/Herdecke, Datteln, Germany; ^4^Vision, Visual Impairments & Blindness, Faculty of Rehabilitation Sciences, Technical University, Dortmund University, Dortmund, Germany; ^5^Developmental Psychology, Faculty of Psychology, Ruhr University Bochum, Bochum, Germany

**Keywords:** associative learning, eyeblink conditioning, extinction, conditioning, renewal, infancy

## Abstract

Associative learning can be observed from the neonatal period onward, providing opportunities to examine changes in basic learning and memory abilities. One method that is suitable to study associative learning is classical eyeblink conditioning (EBC) which is dependent on the cerebellum. Extinction learning can be systematically investigated in this paradigm by varying the context during learning and extinction. Because of methodological difficulties and ethical challenges, no studies have compared extinction learning using EBC across human development. Our goal was to test feasibility of a 3-day delay EBC paradigm that can be used from infancy to adulthood. Acceptance/safety was tested especially for infancy by investigating attrition rates and parental report on infant wellbeing. On a paradigm side, we tested if the paradigm leads to successful acquisition and extinction. An air puff served as unconditional stimulus (US) and a tone as conditional stimulus (CS). On day 1 during acquisition, participants received 36 US–CS pairings in context A. On day 2, participants received 12 acquisition trials in context A to consolidate association learning, followed by 48 extinction trials (tone alone presentations) in context B. Renewal was assessed on day 3 and incorporated 12 CS alone trials presented in both the acquisition context and the extinction context. Eyeblink responses were videotaped and coded offline. The protocol was tested with 12–36-months-old infants (*N* = 72), adolescents (*N* = 8), and adults (*N* = 8). Concerning the acceptance/safety side, attrition ranged from 21 to 58% in infant samples due to the complex preparation of the children for the paradigm. However, attrition is equal to or lower than other infant learning paradigms. Parents of infant samples were very interested in the paradigm and reported low levels of infant stress, exhaustion, and negative feelings during the sessions. Data quality was very high, and no participant had to be excluded because of insufficient data. Concerning the paradigm side, participants showed successful acquisition and extinction as a group. The procedure is ethically sound, feasible, tolerated by many infants, and acceptable among parents. The data show successful acquisition and extinction rates, making the paradigm a valuable tool for investigating developmental changes in extinction learning over the lifespan.

## Introduction

Studying learning and memory across the lifespan not only allow identifying changes in cognitive abilities, but also draw conclusions about the development of underlying neural processes (Jabès and Nelson, 2015). Neural substrates of learning and memory undergo rapid development from early life up until adulthood (Gogtay et al., 2006). Conditioning, extinction, and renewal experiments provide a way of specifically examining the development of cognitive processes and their underlying neural systems. There exist a variety of different conditioning paradigms in animals and human adults. However, there is no paradigm yet suited to investigate conditioning, extinction, and renewal across the human lifespan. Here, we present a study design to fulfill this goal.

After an association is conditioned, it can be extinguished by removing the reinforcer or the unconditioned stimulus (Bouton, 2002). Studies in adults show that extinction does not simply erase the conditioned memory, but inhibits the formerly learned association (Bouton, 2002). Furthermore, extinction learning is context dependent: the extinguished response can return when participants are returned to the context of conditioning. That is, when conditioned in context A, extinguished in context B, the conditional response is shown again when returning to context A (this is called renewal) but not when returning to context B (Bouton and Swartzentruber, 1991). The developmental emergence of renewal in contextual learning seems to depend on the maturation of the hippocampus or hippocampal connections to the cortex (Raineki et al., 2010). Both infant and juvenile rats display extinction, but only juvenile rats show renewal (Rudy, 1993; Rudy and Morledge, 1994; Yap and Richardson, 2007). Specifically, infant rats at postnatal day 17 display complete extinction, whereas rats at postnatal day 21 show renewal (Carew and Rudy, 1991; Kim and Richardson, 2008). These findings point toward a crucial time in rat development for extinction: the transition from infancy to childhood. During that time, the circuitry between hippocampal subfields and cortical–hippocampal connections matures, presumably enabling central aspects of memory function such as contextual learning (e.g., Lavenex and Banta Lavenex, 2013). Some researchers suggest that during human development, the period between 18 and 24 months of age reflects a major milestone in hippocampal development (Gómez and Edgin, 2016).

During adolescence, further changes in extinction might be induced by a mismatch between the maturation of subcortical and prefrontal regions (e.g., Somerville et al., 2010). Studies in both rodent and human adolescents show attenuated extinction (i.e., higher resistance to extinction), as well as heightened renewal (McCallum et al., 2010; Kim et al., 2011; Pattwell et al., 2012). Pattwell et al. (2012) showed diminished extinction in human adolescents (12–17 years) in comparison to both children (5–11 years) and adults (18–28 years) in a fear-conditioning paradigm. Importantly, the differences in extinction between ages were not based on differences in acquisition, which is intact in adolescents. In summary, these results suggest changes in extinction to parallel major transition periods in brain development, that is, a developmentally crucial role of the hippocampus in early extinction and a developmental mismatch of subcortical structures and prefrontal cortex and their circuitry during adolescence (Gee et al., 2013). The developmental changes of extinction might follow a *U*-shaped function.

However, up to now, there are no studies investigating neural development and the associated cognitive processes from infancy to adulthood using the same paradigm, probably due to methodological and ethical problems (Hayne, 2004). First, there are ethical challenges in working with human infant populations. Methods that are usually employed in human adults or animals to assess learning and memory cannot be employed with human infants (Hayne, 2004). Fear conditioning tasks using electro shocks, which are often applied in studies with human adults or animals are unsuitable for infants. Second, tasks that require verbal understanding or production, and complex motor behaviors are unsuitable for infants (Hayne, 2004). Third, in terms of the lifespan approach, developmental changes in interests, attention rates, and physical abilities place limits on the assessment of learning and memory with the same task. Lastly, infant researchers are commonly confronted with high dropout rates. For example, studies using eye-tracking experience dropout rates of around 40–50% due to calibration issues, fussiness, or low data quality (e.g., Amso and Johnson, 2008: 52%; Frank et al., 2009: 47%; Taylor and Herbert, 2013: 38%). Similarly, studies using electroencephalography (EEG) experience high drop-out rates that can range from 30 to 75%, due to refusal to wear the EEG cap, removing the cap during the study, or low data quality (Bell and Cuevas, 2012). Thus, infants mainly drop out due to fussiness or crying during the preparation with the equipment or due to insufficient data quality. We wanted to develop a novel protocol with comparable or lower dropout rates focusing especially on producing high data quality. Together, these points provide major challenges in employing the same paradigm from infancy to adulthood. With the current study, we aim to present methodological details and feasibility data of a learning paradigm suited to assess conditioning, extinction, and renewal over the entire lifespan.

One method that is suitable for studying associative learning and the related memory processes from infancy to adulthood is classical eyeblink conditioning (EBC) (Fitzgerald and Brackbill, 1976; Ivkovich et al., 2002). For example, in a standard delay EBC task, a tone [conditioned stimulus (CS)] is paired with an overlapping air puff to the eye [unconditioned stimulus (US)], which elicits an unconditioned response (UR), a blink. After a certain number of pairings, participants blink before the onset of the air puff and thus show a conditioned response (CR) to the tone. In a standard delay EBC, the tone precedes the air puff and then overlaps with the air puff (i.e., there is a delay between the tone and the air puff onset). Major advantages of the paradigm are that (a) it involves a simple response that does not rely on verbal instructions or complex motor requirements, (b) it involves a defined, highly conserved neural circuitry, (c) it is a non-invasive procedure, (d) it can already be employed with human infants shortly after birth, (e) it can be used to identify biomarkers for neurodevelopmental disorders (Hayne, 2004; Reeb-Sutherland and Fox, 2015). Delay EBC is dependent on the cerebellum (see Gerwig et al., 2007 for a review; Woodruff-Pak et al., 1996). However, by varying the context during learning and extinction, EBC has the potential to study other basic processes across ages, such as context-dependent extinction learning that can that can help to make inferences underlying maturation of the neural circuity of the hippocampus. Given the advantages of the EBC paradigm, it is surprising that there are no studies systematically investigating conditioning, extinction, and renewal across the lifespan with this paradigm. To apply a delay EBC paradigm across development that can fill this gap, a study protocol is needed that can be employed from infancy to adulthood. Here, we present an adaption of a classical delay EBC- protocol and a new study design to fulfill this goal.

To develop such a protocol, there are both technical/practical challenges as well as challenges in the paradigm to ensure successful acquisition and extinction. On a technical/practical side, the setup and entertainment during the procedure have to be considered. In newborns and 1-month-old infants, EBC is usually employed during either sleep or wakefulness using a device taped to the infant’s forehead while the infants lie in a crib (e.g., DeLucia, 1968; Little et al., 1984; Fifer et al., 2010). Even infants at these age groups who were awake during the session did not require any entertainment to complete the session and attrition rate due to excessive crying was very low (e.g., in Little et al., 1984: 1–2 infants out of 64). However, older infants will need to be entertained during the session to maintain cooperation, interest, and attention. It is not possible or ethical to physically restrict them as in the experimental setup used with newborns and 1-month-olds. These difficulties in employing a standard EBC paradigm to infants older than 1 month have been met by Ivkovich et al. (1999, 2002), who adopted the procedure for infants aged 4–5 months. In contrast to studies with younger infants, Ivkovich and colleagues used a special headband with a plastic tube that delivered the air puff. They developed a protocol to maintain the infant’s attention and interest during the procedure using a visual display of different toys shown to the infant while he/she sat on the parent’s lap. In a recent adaption, the setup by Ivkovich and colleagues was applied to12-month-old infants in a delay EBC paradigm (Goodman et al., 2018). In that study, toys and snacks were used to entertain and distract the infants during sessions.

On the side of the paradigm, the parameters for successful acquisition across all age groups have to be considered. Some studies have used the adapted protocol by Ivkovich and colleagues to examine the parameters that are required for infants to show successful conditioning with infants from birth to 5-month of age (Ivkovich et al., 2002; Klaflin et al., 2002; Herbert et al., 2003). These studies typically employed two conditioning sessions 6–8 days apart with 50 acquisition trials per session. Results showed that infants at 4 and 5 months of age required a second acquisition session to successfully show conditioning. Furthermore, it was identified that in delay EBC, the optimal delay between tone and air puff onset for learning changes across development (i.e., the interstimulus interval, ISI). While for 1-month-old infants an ISI of approximately 1,500 ms is optimal, 4–5-months-old exhibit learning at an ISI of 650 ms (Little et al., 1984; Ivkovich et al., 1999; Klaflin et al., 2002). Importantly, at 5 months of age, infants display learning rates comparable to those of adults (Herbert et al., 2003). However, this adapted protocol is only suited for a very small age range, since older infants are less likely to be satisfied by merely visually engaging with moving items and will try to interact directly with them. Thus, whereas the EBC stimulus presentation is effective for use from infancy to adulthood, the entertainment is not. In studies with adults, videos are generally used which could be an appropriate option for older infants as well (e.g., Herbert et al., 2003).

To date only one study assessed extinction learning in an EBC paradigm in infants. Extinction was examined in a study using a small sample size of nine full-term and seven premature infants at 5 months of age (Herbert et al., 2004). After two acquisition sessions 1 week apart, successful learners took part in an extinction session consisting of 50 tone alone trials 1 week later. Results revealed rapid in-session extinction for both full-term and premature infants. However, no study has systematically examined the ontogeny of delay conditioning and extinction learning (Reeb-Sutherland and Fox, 2015).

Only two studies have examined the renewal effect in humans using EBC, both of which studied adults. These studies found opposing results: One was able to show a renewal effect using a standard delay EBC task with an air puff as an US and a tone as a CS (Grillon et al., 2008). The other study did not find a renewal effect but they were utilizing a visual threat EBC task, in which the CR was eye closure, but the CS was a ball moving toward the participant’s head (Claassen et al., 2016). Thus, there is some evidence to suggest that renewal can be successfully shown in a delay EBC task in adults, which makes it suitable for our life-span protocol.

To conclude, studies of human EBC across the lifespan remain limited. Sample sizes in infant studies have been very small, and across-age comparisons are rare. A reason for this is likely to be age-related differences in the experiences that maintain interest and compliance within the session. A standardized visual display of toys as entertainment has been shown to maintain the state of young infants during the session (Ivkovich et al., 2002; Herbert et al., 2003), but may be less suitable for older infants and children. In contrast to Goodman et al. (2018) we found in a pilot study with *n* = 11 infants aged 5–11 months of age that there was low interest in a visual display from around 6 months of age. With the onset of independent locomotion (crawling and walking), infants became reluctant to remain seated on their parent’s lap for an extended time. Therefore, we sought to assess the suitability of using videos for our life-span protocol. The videos used here served two purposes. First, they served to keep the infant, as well as older age groups, engaged but in a passive viewing state during learning. Second, the use of two different videos provided the opportunity for them to serve as a context cue, in addition to features of the room such as illumination colors, for an extinction protocol.

The emphasis of EBC studies in infants has been on examining the conditions and parameters for learning so far. However, EBC has the potential to study other basic processes across ages, such as context-dependent extinction learning that help to make assumptions on the underlying maturation of the neural circuity of the hippocampus. The fact that we can use EBC to measure renewal is one of the biggest advantages and clearly extending its usage beyond prior research. Here we present a new adaption and design of an EBC paradigm that (a) can be employed from infancy until adulthood, and that (b) takes into account the learning environment (i.e., the context) to study extinction and context-dependency. We developed a 3-day delay EBC paradigm and tested feasibility, data quality, and acquisition and extinction rates as a precondition to study renewal with infants, adolescents and adults. The focus of our study was on the infancy period since its application was anticipated to be most difficult in this age group.

## Materials and Equipment

First, we report the stimuli, basic experimental design and paradigm, which is the same across all age groups. Then, we report some age-specific features in the laboratory setup.

### Stimuli

A mildly aversive air puff (1/20 lb/in^2^) served as unconditional stimulus (US) and a 1 kHz 80 dB tone as conditional stimulus (CS). US and CS were chosen in accordance with previous reports (Ivkovich et al., 2002; Klaflin et al., 2002; Herbert et al., 2003). A headband with a flexible plastic tube attached to its left side was used to deliver the air puff to the left eye. For context manipulation, two components were used: room illumination and videos. For the room illumination, two 50W LED lights (Chilitec, Germany) with changeable colors were used. We used the colors red and blue to account for yet undetected red–green color blindness. For the videos, we used Peppa Pig and Timmy the Sheep for infants, and The Planet and The Emperor’s Journey for adolescents and adults. Videos were chosen based on child-friendliness, visual interest, and low arousing content.

### Eyeblink Paradigm

**FIGURE 1:**
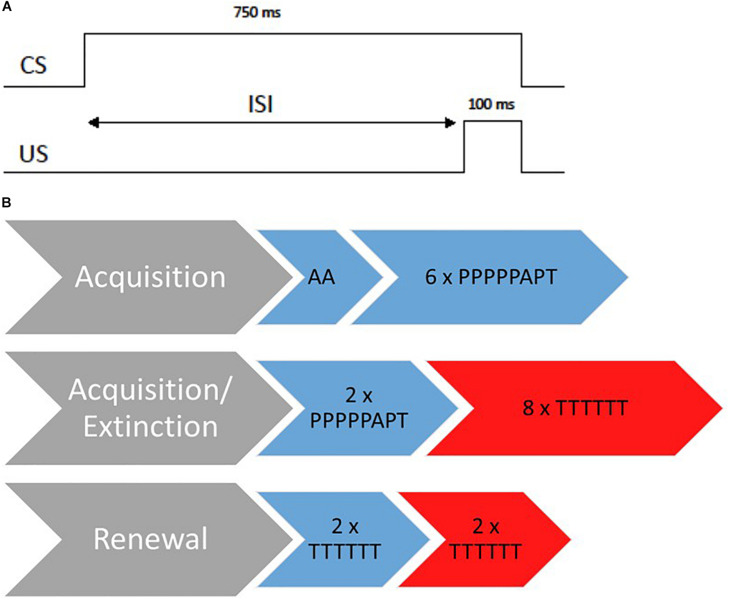
Overview of the 3-day EBC paradigm. **(A)** Standard delay paired trails during acquisition. CS, conditional stimulus; United States, unconditional stimulus; ISI, interstimulus interval. **(B)** Design of the 3-day paradigm. The procedure is displayed for an ABAB design whereas blue stands for the acquisition context and red for the extinction context. A, Air puff alone trials; P, paired trials; T, tone alone trials.

We developed a 3-day delay EBC paradigm that involved three appointments within 14 days. To examine context-dependency in extinction learning, an ABAB/ABBA design was used. On day 1, participants were conditioned during the acquisition session in context A. On day 2, participants were extinguished in context B. On day 3, participants were tested for a renewal effect. Half of the participants were first tested in context A, then in context B. The other half was first tested in context B, then in context A. See Figure 1 for an overview of the paradigm. Context was manipulated by changing the illumination of the room and the video display. A combination of the light and the video served as the acquisition context (context A) and the extinction context (context B). For example, when a participant was conditioned in the blue light/video 1 context, he/she was extinguished in the red light/video 2 context, and then returned to both contexts during renewal (either the acquisition or the extinction context first). There were four possible randomizations between the color and video (blue/video 1; blue/video 2; red/video 1; red/video 2) and participants were randomly assigned to one of the randomizations.

#### Acquisition Session

At the beginning of the acquisition session, there were two air puff alone trials to test responsiveness toward the air puff to correct orientation of the tube. The acquisition session consisted of six blocks of the following trial order: PPPPPAPT (*P* = paired trial, *A* = air puff alone, *T* = tone alone). Paired trials consisted of a 750 ms CS tone overlapping and coterminating with a 100-ms air puff to the eye (see Figure 1; Klaflin et al., 2002). The inter-trial-interval varied from 8 to 16 s. In total, there were 36 paired trials during the acquisition session. Participants had to receive at least 30 paired trails to be included in analyses (Ivkovich et al., 2002). The session lasted approximately 12 min.

#### Acquisition/Extinction Session

Previous studies revealed that infants aged 1–5 months benefit from a second acquisition session and mostly show a significant increase in conditional responses not before finishing another two blocks of acquisition during a second session (Klaflin et al., 2002; Herbert et al., 2003). Therefore, we employed another two blocks of acquisition trials on day 2 (2 × PPPPPAPT) for all age groups in the acquisition context.

Directly afterward, all participants were extinguished in context B for 48 tone alone trials (8 × TTTTTT). The whole session on day 2 lasted approximately 16 min.

#### Renewal Session

Renewal was assessed on day three and incorporated two blocks of tone alone trials (2 × TTTTTT) presented both within the acquisition context and within the extinction context (order of context was counterbalanced across participants). The session lasted approximately 6 min.

### Laboratory Setup

**FIGURE 2:**
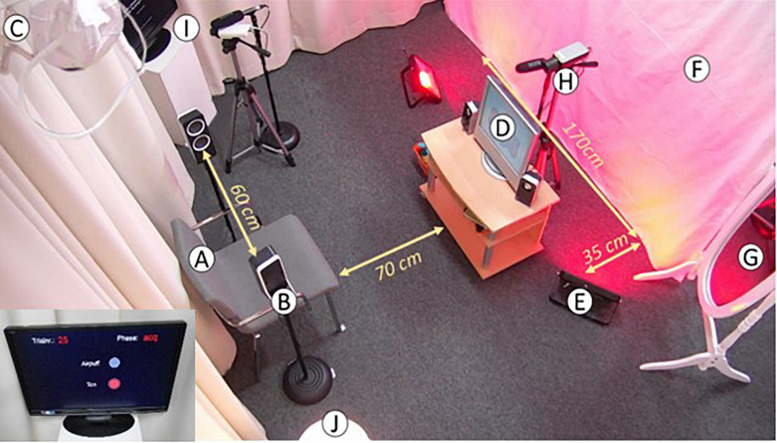
Overview of the setup in the testing room. **(A)** Chair on which caregiver and infant are seated. **(B)** Two speakers to deliver the tone. **(C)** Headband. **(D)** Screen to display the video. **(E)** Two LED lights to illuminate the wall. **(F)** White cloth that is illuminated in different colors depending on context. **(G)** Mirror where caregiver can monitor infant. **(H)** Camera filming the infant’s head to record eyeblinks. **(I)** Camera filming the paradigm that displays the trial. **(J)** Lamp to illuminate the room.

The experiment took place in a laboratory that consisted of a control room and a testing room. The control room was used as the location for explaining the study to the participants/warm-up playroom and the other room was the testing room (see Figure 2 for an overview of the testing room). The video was shown approximately 1 m in front of the participant on a 19 inch screen. Two 50W LED lights (Chilitec, Germany) illuminated a wall in either blue or red. The ceiling light was switched off during the sessions to increase intensity of the context light. The experimenter was able to pause the paradigm if the participant moved and the eye was not visible on camera, or if the air tube/headband needed adjusting. The same experimenter conducted all three sessions with one participant to avoid social context variation (Learmonth et al., 2005).

#### Infants

Infants sat on the parent’s lap during testing. As Ivkovich et al. (2002) suggested, a mirror was placed approximately 2.5 m to the right hand side of the chair so parents could monitor or interact with their infant without the infant having to turn around and thus blocking view of their eye. An additional EEG cap was used to keep the headband fixed at the infants head during the experiment. CS were delivered to the infants through two 8-ohm speakers mounted on the infant’s ear level. To increase visibility of the infant’s eye on the video, a lamp was positioned in the corner of the room on the right hand side of the child. The experimenter sat on the right hand side of the video screen within reach of the infant in case the headband or air tube was out of place. Parents were instructed to put their arms over their infant’s arms to prevent them from grabbing the headband or air tube. The experimenter had two hand puppets and building blocks at hand, in case the infants became unsettled or lost interest in the video. The experimenter abstained from making quick movements to avoid eliciting eyeblink responses and only moved the toys slowly between trials. The screen displaying the paradigm was facing toward the experimenter for this reason.

#### Adolescents and Adults

During testing, participants sat in the testing room by themselves, without a parent or the experimenter present, facing a table where the video was shown on a laptop. CS were delivered through headphones.

### List of Equipment

• For all age groups: headband with flexible plastic tube (headband: supercope, Kikutani Inc., Japan) Plastic tube: loc-line flexi, IBT Thiermann, Germany,
• Additionally for infants: EEG cap to fixate headband,
• Compressor for air puff,
  ∘ Pump: Inqua, (Inqua GmbH, Germany) flow min 3.01/min, flow max 6.017 min, max. pressure 29 psi (0.5bar),
  ∘ 6-mm tubes,
  ∘ Flow level meter to reduce flow,
  ∘ Solenoid valve (combined with pressure release valve, which releases pressure when solenoid valve is closed),
  ∘ Solenoid valve switched by parallel port of presentation computer,
  ∘ Pressure meter to calibrate flow,
• Two 8-ohm speakers/headphones,
• 2 cameras with high frame rate,
• Mirror,
• Screen to display trial,
• Screen/Laptop to show a video,
• Age-based videos that are not arousing,
• Two 50W LED lights (Chilitec, Germany) with changeable colors,
• Lamp to illuminate test room.

## Methods

The method was implemented with a sample of infants, adolescents, and young adults. The objective was to evaluate feasibility and the effectiveness of this new protocol for gaining quality data, as well as showing that the protocol leads to successful acquisition and extinction. Since the application of the EBC paradigm was anticipated to be most difficult in infants, we focused on the infancy period in this study and tested a large sample of infants. The study was approved by the ethics committee of the department of psychology at Ruhr-University Bochum.

### Participants

Families were recruited using local birth registers. Exclusion criteria were hearing or vision problems or preterm birth. Families received 10 € and a small gift for the child per session. Seventy-two infants from 12 to 36 months of age (*M*_*age*_ = 651 days; *SD*_*age*_ = 263; range = 348–1,241 days; *n* = 33 female) participated in the study (*n* = 33 in condition ABAB; *n* = 39 in condition ABBA).

Adolescents were recruited using flyers, community events or word of mouth. Exclusion criteria were hearing problems, neurological disorders or color blindness. Participants with vision problems could wear glasses during the session and were not excluded. They received 10 € worth of gift cards per session. Eight adolescents aged (*M*_*age*_ = 13.4 years; *SD*_*age*_ = 1.5, range = 12–16 years; *n* = 4 female) participated in the study (*n* = 6 in condition ABAB; *n* = 2 in condition ABBA). Adult participants were students recruited at university using Listserv and word of mouth. Participants with vision problems could wear glasses during the session and were not excluded. However, contact lenses were not permitted, since participants could be less sensitive to the air puff when wearing them. Exclusion criteria were hearing problems, neurological disorders, or color blindness. Eight adults (*M*_*age*_ = 21.3 years; *SD*_*age*_ = 2.4; range = 18–26 years; *n* = 5 female) participated in the study (*n* = 6 in condition ABAB; *n* = 2 in condition ABBA). They received course credit or 10€ worth of gift cards per session.

### Questionnaires

#### Feasibility Assessment

In contrast to adolescents and adults, the same EBC protocol has never been applied with infants ranging from 12 to 36 months of age (see Goodman et al., 2018, for the application in 12-month-old). Thus, feasibility was assessed to capture how parents rate their infant’s as well as their own stress during the sessions. To assess feasibility in the infant sample, parents completed a questionnaire on a tablet following each session. We asked how the mother perceived infant’s stress and feelings during the session on a 10-point Likert scale from “not at all” to “very” (i.e., “How exhausting was the session for your child?”; “How strong were his or her negative feelings during the session?”). We also asked how the mother perceived the session on a 10-point Likert scale from “not at all” to “very” (“How interesting was the session for you?”; “How exhausting was the session for you?”). Additionally, parents indicated after session 3 if they would participate in the study again.

#### Infant Temperament

To compare infants who drop out with infants who did not drop out from the study, parents filled out an age-appropriate questionnaire on their infant’s temperament. For 12-month-old infants, the 37-item Infant Behavior Questionnaire (IBQ) was used (Putnam et al., 2014). For 18–36-months-old infants, the 36-item Early Child Behavior Questionnaire (EBCQ) was used (Putnam et al., 2006). Parents rate if their child shows a certain behavior over the last 2 weeks on a 7-point Likert scale ranging from “1 = never” to “7 = always.” The questionnaires can be scored into three scales capturing dimensions of temperament: surgency/extraversion, negative affectivity, and effortful control.

### Procedure

Participants were randomly assigned to one of the two conditions (ABAB/ABBA) before arrival at the laboratory. At the beginning of session 1, the experimenter explained the study and procedure and obtained written consent from participants and/or parents.

The procedure during each of the three sessions was similar. There was a 10 min warm-up period involving playing with toys in the front part of the room so the infant had time to get comfortable and get used to the surroundings and the experimenter. For adolescents and adults, the experimenter engaged in small talk to make the participant feel comfortable. Then, the experimenter, the parent, and the infant (or the participant alone for adolescents and adults) moved behind the room divider to the testing room. The second experimenter moved to the control room to start video recordings, control the cameras, and to start the paradigm. The experimenter attached the headband and adjusted the flexible tube. When ready, the experimenter switched off the light, turned the colored context illumination on, and started the video display. Then, the second experimenter first started video recordings and then the paradigm once the participant was settled and focused on the video display. At the end of the paradigm, the headband was removed, the video was stopped and the illumination was switched off. Participants and experimenter moved to the front part of the room again. After each session, parents of infant participants filled out the feasibility questionnaire on a tablet. At the end of each session, the participant received a small present/gift card/course credit. Participants who did not complete session 1 were not invited to sessions 2 and 3.

### Data Reduction

As recommended by Goodman et al. (2018), videotaped sessions were coded offline frame-by-frame using the software INTERACT (Mangold International GmbH, Arnsdorf, Germany) for the occurrence of eyeblinks by trained coders. A second coder countercoded videos of 32 participants for analysis of interrater reliability. The coding scheme developed by Herbert and colleagues was employed: For paired trials, a conditional response (CR) was coded if the eyeblink occurred 300 ms after tone onset but before the onset of the air puff. Eyeblinks within 300 ms after tone onset were coded as a startle response. An eyeblink occurring within 500 ms after air puff onset was coded as a UR. For tone alone trials, eyeblinks were coded as a CR when occurring 300 ms after tone onset until the UR period (see Herbert et al., 2004). This longer time window for CRs in tone alone trials was chosen to being able to compare to previous studies in infants (e.g., Klaflin et al., 2002; Herbert et al., 2004). Furthermore, previous infant studies showed that results did not differ when CRs during tone alone trials were coded as blinks within 300 ms after tone onset compared to CRs coded as adaptive responses (i.e., eyeblinks within 350 ms prior to air puff onset) in a 650 ms delay paradigm (Klaflin et al., 2002). With the longer time window, we want to account for the possibility that some infant age groups might not be able to time the CRs very well (Ivkovich et al., 2002; Herbert et al., 2004).

For the acquisition session, %CRs were calculated for blocks of six paired trials (eight blocks in total). Additionally, %CRs were calculated for the eight tone alone trials. To investigate UR to the air puff, %UR were calculated for the 10 air puff alone trials. For the extinction and renewal sessions, %CR were calculated for tone alone trials for blocks of six trials (eight blocks for extinction, two blocks for each context during renewal). For trials where the participant’s eye was not visible, they were classified as not codable. The percentage of not codable trials was used as a measure of data quality. For a block to be included into the analyses, participants had to have at least three codable trials out of six. In the rare instances where a block was not codable, the value was replaced by the participant’s mean %CR of the previous and subsequent block. If the missing block was the first or the last block, the mean value of the age groups %CR for this block was used (see Herbert et al., 2003).

Individual learning and extinction rates were calculated as well. Trials-to-criterion (TTC) is a measure of how many trials each participant needs to fulfill the learning criterion during acquisition (three CRs out of a block of six paired trials; Ivkovich et al., 2002). If a participant failed to fulfill the learning criterion, he/she was given a score of 58 (the maximum number of trials plus 10; Ivkovich et al., 2002). We adopted this criterion to the extinction session to measure how many trials each participant needs to reach the extinction criterion (less than three CRs out of six, a block of six tone alone trials). If a participant failed to fulfill the extinction criterion, he/she was given a score of 58 (the maximum number of trials plus 10).

### Data Analyses

#### Feasibility

##### Dropout rates

Dropout rates per session and age group were analyzed.

##### Feasibility across sessions

Infants who completed all three sessions were analyzed on the items of the feasibility questionnaire across sessions using repeated-measures ANOVAs to assess changes in stress. Pairwise comparisons using paired *t* tests with Bonferroni correction were conducted to determine which sessions significantly differ from each other. To assess systematic dropout, we divided the infant sample in completers and non-completers of session 1 and compared them on infant temperament and measures of socioeconomic status (SES) with *t* tests for independent samples.

##### Feasibility in completers and non-completers of session 1

To capture infant’s state who only participated in session 1, we divided the infant sample in completers and non-completers of session 1 and compared them on the items of the feasibility questionnaire with *t* tests for independent samples.

#### Acquisition

##### Paired trials

The data from infants, adolescents and adults was analyzed together. The data from all participants who completed acquisition was analyzed, regardless of whether or not they reached the TTC. To analyze if participants showed acquisition as a group, statistical analyses of CR were conducted as a repeated-measures ANOVA with block (8) as a within-subject factor. Pairwise comparisons using paired *t* tests with Bonferroni correction were conducted to determine which blocks significantly differ from each other.

##### Tone alone trials

Individual %CR in tone alone trials were analyzed.

##### Trials-to-criterion

To assess individual learning rates, TTC scores were analyzed.

##### Responsiveness to air puff

To analyze if responsiveness to the air puff is related to conditionability, mean %UR was correlated to TTC and %CR in tone alone trials.

#### Extinction

To study extinction, only the data from participants who showed successful acquisition as indicated by individual TTC scores was analyzed. To analyze if participants showed extinction as a group, statistical analyses of CR were conducted for eight blocks of extinction with a repeated-measures ANOVA. Pairwise comparisons using paired *t* tests with Bonferroni correction were conducted to determine which blocks significantly differ from each other. To analyze if participants generalized across the acquisition and extinction context, a paired *t* test on the CRs during the last acquisition block and the CRs during the first extinction block was conducted. A non-significant paired *t* test will suggest a complete generalization of acquisition (Vervliet et al., 2013).

##### Trials-to-criterion

To assess individual extinction learning rates, TTC scores were analyzed.

## Results

### Data Quality

Interrater reliability was excellent, intra-class correlation coefficient = 0.987. For the infant sample, 34 paired trials (1.7%), 45 tone alone trials (2.1%), and eight air puff alone trials (1.9%) were not codable. For the adolescent sample, only two paired trials (0.5%) and two tone alone trials (0.35%) were not codable. All air puff alone trials were codable. For the adult sample, all paired trials, all tone alone trials, and all air puff alone trials were codable. Thus, in no case, a block had to be replaced by the group mean or the mean of the previous and subsequent block.

### Feasibility

#### Dropout

There were no adolescents or adults who dropped out of the study at any point. In the infant sample, *n* = 43 out of 72 infants completed the acquisition session. The other *n* = 29 did not complete session 1 and had to be excluded due to crying (*n* = 17), or not tolerating the headband (*n* = 12). Forty-one infants completed the extinction session and 40 infants completed the renewal session. See Table 1 for the number of infants per age group that completed each session and for dropout reasons. For the infant sample, dropout rates in session 1 were 43% in 12-month-olds, 61% in 18-month-olds, 32% in 24-month-olds and 21% in 36-month-olds (see Table 1). Infants either dropped out during session 1 due to fussiness and crying (59%), or refusal to wear the headband (41%). Generally, if children completed session 1, they were very likely to complete sessions 2 and 3. Only two children dropped out in session 2 due to falling asleep during the extinction session and due to refusal to wear the headband. Parents of one child canceled session 3 due to sickness.

**TABLE 1:** Number of participants per age group that participated (first rows) and that completed (second rows) each session, including reasons for exclusions.

**Age group**	**Acquisition session**	**Extinction session**	**Renewal session**
12-month-olds	*n* = 21	*n* = 12	*n* = 10
	Completed: *n* = 12	Completed: *n* = 11	Completed: *n* = 10
	Excluded: *n* = 2 not tolerating headband; *n* = 7 crying	Excluded: *n* = 1 fell asleep	Excluded: *n* = 1 canceled appointment due to sickness
18-month-olds	*n* = 18	*n* = 7	*n* = 6
	Completed: *n* = 7	Completed: *n* = 6	Completed: *n* = 6
	Excluded: *n* = 6 not tolerating headband; *n* = 5 crying	Excluded: *n* = 1 not tolerating headband	
24-month-olds	*n* = 19	*n* = 13	*n* = 13
	Completed: *n* = 13	Completed: *n* = 13	Completed: *n* = 13
	Excluded: *n* = 1 not tolerating headband; *n* = 5 crying		
36-month-olds	*n* = 14	*n* = 11	*n* = 11
	Completed: *n* = 11	Completed: *n* = 11	Completed: *n* = 11
	Excluded: *n* = 3 not tolerating headband		
Adolescents	*n* = 8	*n* = 8	*n* = 8
Adults	*n* = 8	*n* = 8	*n* = 8
Total	*N* = 88	*N* = 59	*N* = 56
	Completed: *n* = 59	Completed: *n* = 57	Completed: *n* = 56

Infants who dropped out did not significantly differ in temperament as assessed by a temperament questionnaire from infants who did not drop out in session 1 [surgency/extraversion: *t*(49) = −0.12, *p* = 0.907; negative affectivity: *t*(49) = −0.16, *p* = 0.875; effortful control: *t*(49) = 0.06, *p* = 0.951; *n* = 18 parents of non-completers and *n* = 33 parents of completers filled out the questionnaire.] Furthermore, as measures of SES, parents of completers and non-completers did not differ in their level of education [maternal education: *t*(25.41) = −1.06, *p* = 0.301, paternal education: *t*(49) = −1.22, *p* = 0.227], or in the family’s net income after taxes, *t*(49.93) = 1.00, *p* = 0.322. Thus, dropout does not seem to be systematic.

#### Feasibility Across Sessions

**FIGURE 3:**
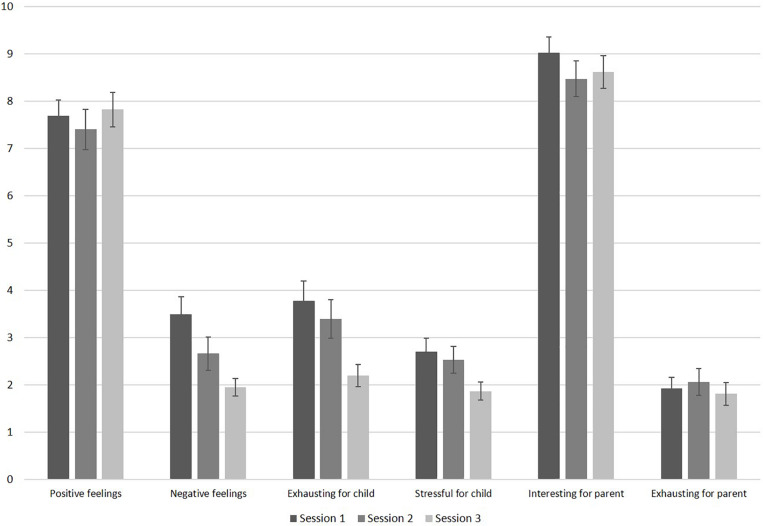
Results of the feasibility questionnaire filled out by parents after each session (sample sizes: positive feelings: *n* = 34; negative feelings: *n* = 35; all others: *n* = 36). The scale ranges from 10 (very high) to 0 (very low). Error bars are SE of M.

Parents indicated low levels of infant exhaustion and stress during session 1. Figure 3 displays the results of the feasibility questionnaire. Parents’ ratings on how exhausting the session was for their infant significantly decreased throughout the sessions, *F*(2, 70) = 7.63, *p* = 0.001, η_*p*_^2^ = 0.179. Pairwise comparisons revealed significant differences in infant exhaustion between sessions 1 and 3 (*p* = 0.004), and between sessions 2 and 3 (*p* = 0.012). Furthermore, parental report on infant levels of stress decreased significantly throughout the sessions, *F*(2, 70) = 4.18, *p* = 0.019, η*_*p*_*^2^ = 0.107. Pairwise comparisons revealed significant less infant stress between sessions 1 and 3 (*p* = 0.024). Infants’ negative feelings also decreased significantly throughout the sessions, *F*(1.55, 52.83) = 8.08, *p* = 0.002, η*_*p*_*^2^ = 0.192 with significantly less reported negative feelings between sessions 1 and 3 (*p* = 0.001), and between sessions 2 and 3 (*p* = 0.040). Infant’s positive feelings remained high throughout the sessions, *F*(1.57, 51.80) = 0.45, *p* = 0.590, η*_*p*_*^2^ = 0.014. As visible in Figure 3, parents indicated that their levels of exhaustion was low throughout all sessions. Their level of exhaustion did not change across sessions, *F*(1.65, 57.69) = 0.37, *p* = 0.652, η*_*p*_*^2^ = 0.010. Parents found the study interesting throughout all sessions (see Figure 3) and the level of interest did not change across sessions, *F*(2, 70) = 2.08, *p* = 0.133, η*_*p*_*^2^ = 0.056. If parents completed all three sessions, they were asked whether they would participate in this study again. Of the 24 parents who answered this question, all responded “yes” (*n* = 13 did not provide this information).

#### Feasibility in Completers and Non-completers of Session 1

**FIGURE 4:**
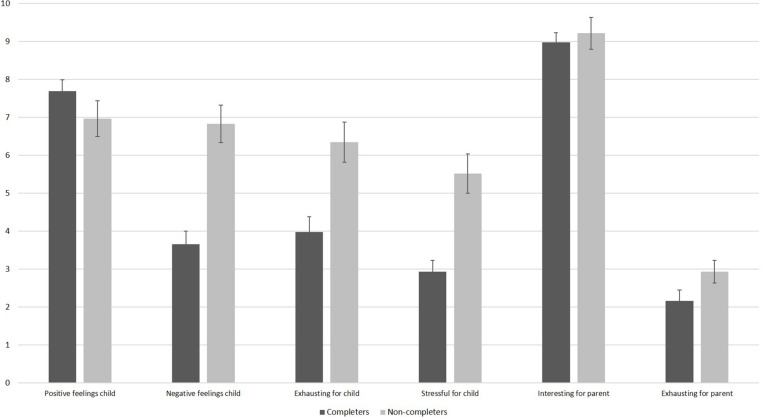
Results of the feasibility questionnaire filled out by parents after session 1 as a function of completers and non-completers. *N* = 42 parents of completers provided answers for all items, except for negative feelings where only 41 parents provided answers. One parent of a completer did not provide any information. *N* = 29 parents of completers provided answers for the child items, and 28 parents of completers provided answers for the parent items. The scale ranges from 10 (very high) to 0 (very low). Error bars are SE of M.

We divided infants into completers of session 1 and non-completers of session 1 and compared the parents’ responses on the feasibility questions (see Figure 4). As visible, even parents of non-completers only rated moderate levels of infant stress and exhaustion. Parents of non-completers reported higher levels of exhaustion, *t*(69) = −3.62, *p* = 0.001, stress, *t*(45.88) = −4.31, *p* < 0.001, as well as higher negative feelings in their infant than parents of infants who completed session 1, *t*(68) = −5.46, *p* < 0.001. There was no significant difference regarding positive feelings during the session between completers and non-completers, *t*(69) = 1.37, *p* = 0.177. As shown in Figure 4, parents of completers and non-completers were both highly interested in the study. They did not differ in how interesting the study was for them, *t*(68) = −0.56, *p* = 0.575, or in how exhausting the session was for them, *t*(45.97) = −1.57, *p* = 0.124.

### Eyeblink Paradigm

To test whether our protocol produced usable data for a future application, we analyzed if participants as a group (infants, adolescents, and adults) successfully acquired and extinguished the association between tone and air puff as a precondition to investigate renewal in future studies.

#### Acquisition

**FIGURE 5:**
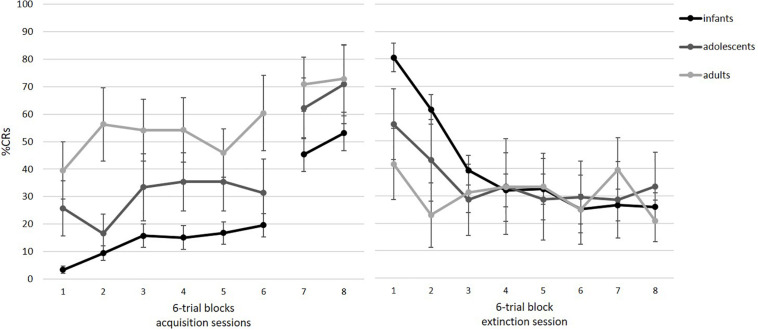
Mean percent CRs for the acquisition **(left)** and extinction session **(right)** as a function of age group. Acquisition consisted of six blocks in session 1 and two blocks at the beginning of session 2. Acquisition: infants: *n* = 42, adolescents: *n* = 8, adults: *n* = 8. Extinction: infants: *n* = 26, adolescents: *n* = 7, adults: *n* = 8. Error bars represent SE of M.

Participants successfully acquired the association between the tone and the air puff. Note that only those participants who also completed the two acquisition blocks from session 2 could be included. Figure 5 illustrates acquisition rates for eight blocks of six paired trials. As shown, there is an increase in %CR, which was supported by a repeated-measures ANOVA with block as a within-subject factor, *F*(3.73, 212.31) = 32.87, *p* < 0.001, η*_*p*_*^2^ = 0.366. The significant increase was especially visible from the end of session 1 (block 6) to session 2 on day 2 (blocks 7 and 8). Pairwise comparisons revealed significant differences between blocks 1–6 and blocks 7–8 (all *p*’*s* < 0.05). Furthermore, we analyzed in how many of the eight tone alone trials participants showed a conditional response as an additional measure of acquisition. Participants showed an average of 48% CRs (*SD* = 34%; range = 0–100%).

**FIGURE 6:**
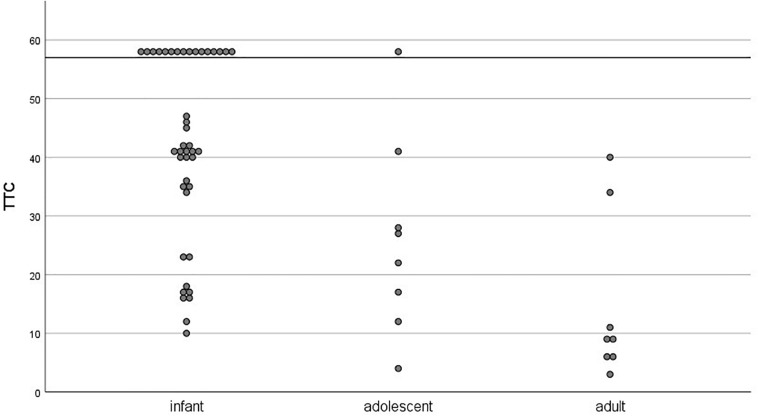
Individual trials-to-criterion (three CRs out of a block of 6 paired trials) for the acquisition session as a function of age group. Participants who did not reach criterion during the acquisition session (48 paired trials) were assigned a score of 58 and are visible above the horizontal line.

On an individual level, we tested how many participants per age group reached the TTC learning criterion during acquisition session (three CRs out of a block of six paired trials). There was considerable individual variance in TTC scores within each age group (see Figure 6). All age groups but adults contained learners and non-learners (infants: 62% learners; adolescents: 88% learners). Only learners were considered in the extinction analyses.

Last, we analyzed responsiveness toward the air puff (%UR during air puff alone trials). Overall, participants were very responsive toward the air puff with a mean %UR of 93% (*SD* = 12%) and a range from 50 to 100%. To analyze if responsiveness to the air puff is related to conditionability, mean %UR was correlated to TTC and %CR in tone alone trials. %UR was marginally significantly related to TTC score (*r* = −0.229, *p* = 0.084, *N* = 58) and %CR tone alone trials (*r* = 0.239, *p* = 0.071, *N* = 58).

#### Extinction

Figure 5 illustrates extinction rates across eight blocks of six tone alone trials. As visible, there is a decrease in %CR across session, indicating extinction of the association between the tone and the air puff. As a group, participants showed successful extinction, as revealed by a significant repeated measures ANOVA, *F*(4.22, 168.96) = 22.41, *p* < 0.001, η*_*p*_*^2^ = 0.359. Pairwise comparisons indicated that the significant decrease occurred from block 1 to 2 (*p* < 0.001). Furthermore, participants generalized across acquisition and extinction context, *t*(40) = 1.36, *p* = 0.182, *η*_*p*_^2^ = 0.044,.

On an individual level, we tested if participants showed extinction as indicated by the TTC criterion during extinction session (less than three CRs out of a block of six paired trials). All participants except for two infants and one adolescent reached extinction until the end of the session. There was considerable individual variance in TTC scores during extinction within each age group (infants: *M* = 21, *SD* = 14, *n* = 26; adolescents: *M* = 20, *SD* = 20, *n* = 7; adults: *M* = 9, *SD* = 5, *n* = 8).

#### Renewal

**FIGURE 7:**
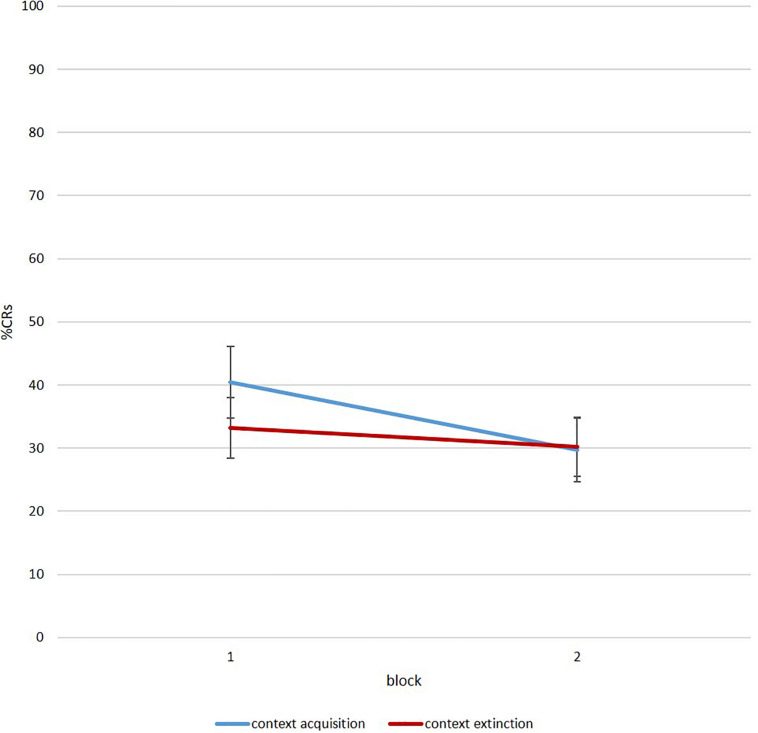
Mean percent CR for six-trial blocks during renewal in the acquisition (blue) and extinction context (red) (*N* = 40 of which *n* = 25 infants and adolescents/adults: *n* = 15).

To investigate a renewal effect with this protocol, participants need to show some eyeblink reactions to the tone during session 3. Figure 7 illustrates %CR for acquisition and extinction context for all participants as a group. As visible, the paradigm created valid data in order to analyze the emergence of renewal during ontogeny in future studies.

### Anticipated Results of the Protocol in Future Studies

The presented study shows that the protocol presented here can be applied with all age groups between 12 months and adults. The paradigm is safe for infants and well accepted by their parents. In future studies, the protocol can be used to study age differences in extinction and renewal across life span and thus, improve our understanding of learning and memory and its underlying maturation of neural structures such as the hippocampus.

#### Acquisition

We expect age differences in acquisition rates in that adolescents and adults will acquire the association between the tone and the air puff faster than infants (Herbert et al., 2003). Furthermore, we expect higher acquisition rates, the older the children (Löwgren et al., 2017).

#### Extinction

Following the developmental mismatch model, we hypothesize that the maturing PFC influences extinction and renewal. We expect that adolescents show attenuated extinction in contrast to adults (Somerville et al., 2010; Kim et al., 2011). Taking a closer look at adolescence, the developmental changes of extinction might follow U-shaped function with its dip in mid adolescence (Pattwell et al., 2012). Because of missing literature on extinction learning in infants, precise hypotheses on the development of quantitative markers of extinction (e.g., time-to-extinction) are not formulated.

#### Renewal

We expect that younger infants will show no renewal in contrast to older infants/children, adolescents and adults. The exact time point for the emergence of renewal is assumed between 18 and 24 months of age (Gómez and Edgin, 2016). Furthermore, we expect that adolescents show attenuated renewal in contrast to adults. The developmental changes of extinction might follow a U-shaped function with its dip in mid adolescence.

## Discussion

We sought to develop a new 3-day EBC paradigm that can be used to study associative learning, extinction, and renewal from infancy to adulthood. Despite several methodological and ethical challenges, we employed a delay EBC paradigm with the same parameters for infants from 12 months of age to adulthood. While the main features of the protocol are the same for all age groups, we made age-specific adjustments such as the entertainment for the infant sample and used child-friendly videos and the parent being present during testing. Finally, we embedded two context variables to be able to study context-dependent extinction learning. Results indicate successful acquisition and extinction, which is a precondition to study renewal in the future.

We found that infants experienced low levels of stress, negative feelings, and exhaustion during the sessions as measured through parental questionnaires. Moreover, these measures decreased from session1 to session 3. Even in non-completers of session 1, parents reported only moderate levels of stress. Parents were highly interested in the paradigm regardless of whether their infant completed the paradigm or not, thus showing that the paradigm is accepted by parents of this young age group.

We experienced the highest dropout rates in infant samples in the first session. Dropout rates were very low once the child completed session 1. Dropout rates in session 1 were higher in 12- and 18-month-olds olds compared to previous studies using EBC with 4- and 5-month-old children (Ivkovich et al., 2002; Herbert et al., 2003), but comparable to an EBC study in 12-month-olds (Goodman et al., 2018). In 24-month-old, dropout rates were approximately equal to previous studies. In 36-month-olds, they were lower compared to previous studies using EBC with 4- and 5-months-old children (Ivkovich et al., 2002; Herbert et al., 2003). Thus, as a general rule, the protocol is more feasible the older the infant. Previous infant studies using EBC experienced around 1/3 of non-completers (Ivkovich et al., 1999; Klaflin et al., 2002). Higher dropout rates in some of our age groups could be explained because infants in previous studies were much younger and thus limited in motor abilities which makes the paradigm easier to conduct (Little et al., 1984; Ivkovich et al., 1999; Klaflin et al., 2002). Compared to other paradigms in infant studies such as eye-tracking (e.g., Amso and Johnson, 2008; Frank et al., 2009) and EEG (Bell and Cuevas, 2012), we experienced equal to lower dropout rates. Furthermore, infants in the present study dropped out for the most part due to refusal to wear the headband. This fussiness during preparation with equipment is common in infant studies and thus not specific for our protocol, and most likely would have led to termination also in any other experimental setting. Hence, the large proportion of infants dropped out while preparing for the EBC paradigm and not due to the paradigm itself or the air puff. Compared to other commonly employed infant paradigms such as eye-tracking and EEG, our paradigm had no attrition due to low data quality. When a participant completes the paradigm, data quality is very high and only in a few cases, a trial was not codable.

First application of the protocol in infants, adolescents, and adults shows successful acquisition rates across all age groups. Acquisition levels were quite low and only gradually increased in session 1 but significantly increased and peaked in session 2. Thus, a second acquisition session with two additional blocks of paired trials on day 2 of the paradigm are both necessary and sufficient to demonstrate robust acquisition. Acquisition levels were similar to those observed in infants and adults using 650 ms delay EBC and human adults (Ivkovich et al., 2002; Herbert et al., 2003).

Furthermore, individual learning rates during acquisition (TTC) showed high variability between participants. This variability in acquisition rates can be valuable in order to relate to age-related differences and social and cognitive outcomes (Reeb-Sutherland et al., 2012), as well as neurodevelopmental disorders such as autism spectrum disorder (Reeb-Sutherland and Fox, 2015). This has to be tested in future studies with bigger samples.

Following acquisition, participants exhibited successful extinction of learned responses as a group. The number of blocks during extinction was sufficient to ensure an overall decrease in %CRs. Furthermore, an individual extinction learning criterion was successfully applied and can possibly be linked to developmental outcomes and neurodevelopmental disorders in the future.

The EBC-paradigm also allows including several other control conditions. First, an unpaired conditioning control condition could be included to control for non-associative increases in response to the tone. Second, additional renewal designs can be easily added, such as an AAA control condition to control for spontaneous recovery. Thus, there are some options to easily vary and add conditions to this paradigm. Furthermore, electromyography (EMG) could be added as an additional measure of eyeblinks in older children and adults. This would also give the opportunity for a more detailed analysis of response behavior generally reported in adults such as response amplitude and response latency. We abstained from including EMG in our infant sample since it leads to even higher attrition rate in our experience.

Studying renewal across the lifespan can be especially intriguing since it allows conclusions on underlying maturation of neural structures such as the hippocampus, which has been a long-standing debate (Jabès and Nelson, 2015; Gómez and Edgin, 2016). Our data show successful application of the paradigm, which gives us the opportunity to study renewal in future studies with adequate powered sample sizes. To our knowledge, the current research design will be the first to provide a systematic account of extinction in infancy/early childhood and adolescence. Furthermore, using the presented EBC protocol it is possible to translate findings in rodents on qualitative changes in extinction learning during infancy to humans. Understanding the developmental trajectories of extinction not only provides insight into the mechanisms underlying extinction, but has direct clinical implications in the field of clinical child and adolescent psychology, potentially translating windows of vulnerability into windows of opportunity.

In summary, despite multiple methodological and ethical challenges, we were able to develop an EBC protocol that is ethically sound, feasible, tolerated by many infants, and acceptable among parents. The EBC paradigm is identical for all-age groups except for some relatively minor age-specific adaptations in the entertainment, videos, and laboratory setup. The 3-day EBC protocol thus provides a valuable tool to investigate developmental changes in associative and extinction learning from infancy to adulthood.

## Data Availability Statement

The datasets presented in this article are not readily available because: The dataset presented in this article is a subset of a bigger study and will be made available by the authors after completion of the study. Requests to access the datasets should be directed to CK, carolin.konrad@rub.de.

## Ethics Statement

The studies involving human participants were reviewed and approved by Ethics Committee of Department of Psychology, Ruhr University Bochum. Written informed consent to participate in this study was provided by the participants’ legal guardian/next of kin.

## Author Contributions

SSc conceived the study idea. SSc, DA, CM, JH, SW, and CK contributed to the conception and design of the study. DA built the apparatus. CK, LN, and JJ-P collected the data. CK performed the statistical analysis and wrote the manuscript. All authors contributed to manuscript revision, read and approved the submitted version.

## Conflict of Interest

The authors declare that the research was conducted in the absence of any commercial or financial relationships that could be construed as a potential conflict of interest.
